# Supplementary figures and images for: A Genome-Wide Association Study for Host Resistance to Ostreid Herpesvirus in Pacific Oysters (Crassostrea gigas)
Source: G3 (Bethesda). 2018 Feb 22;8(4):1273–80. doi: 10.1534/g3.118.200113 (PMC5873916; doi:10.1534/g3.118.200113)

Figure S1. Length distribution of scaffolds according to the number LGs they were assigned


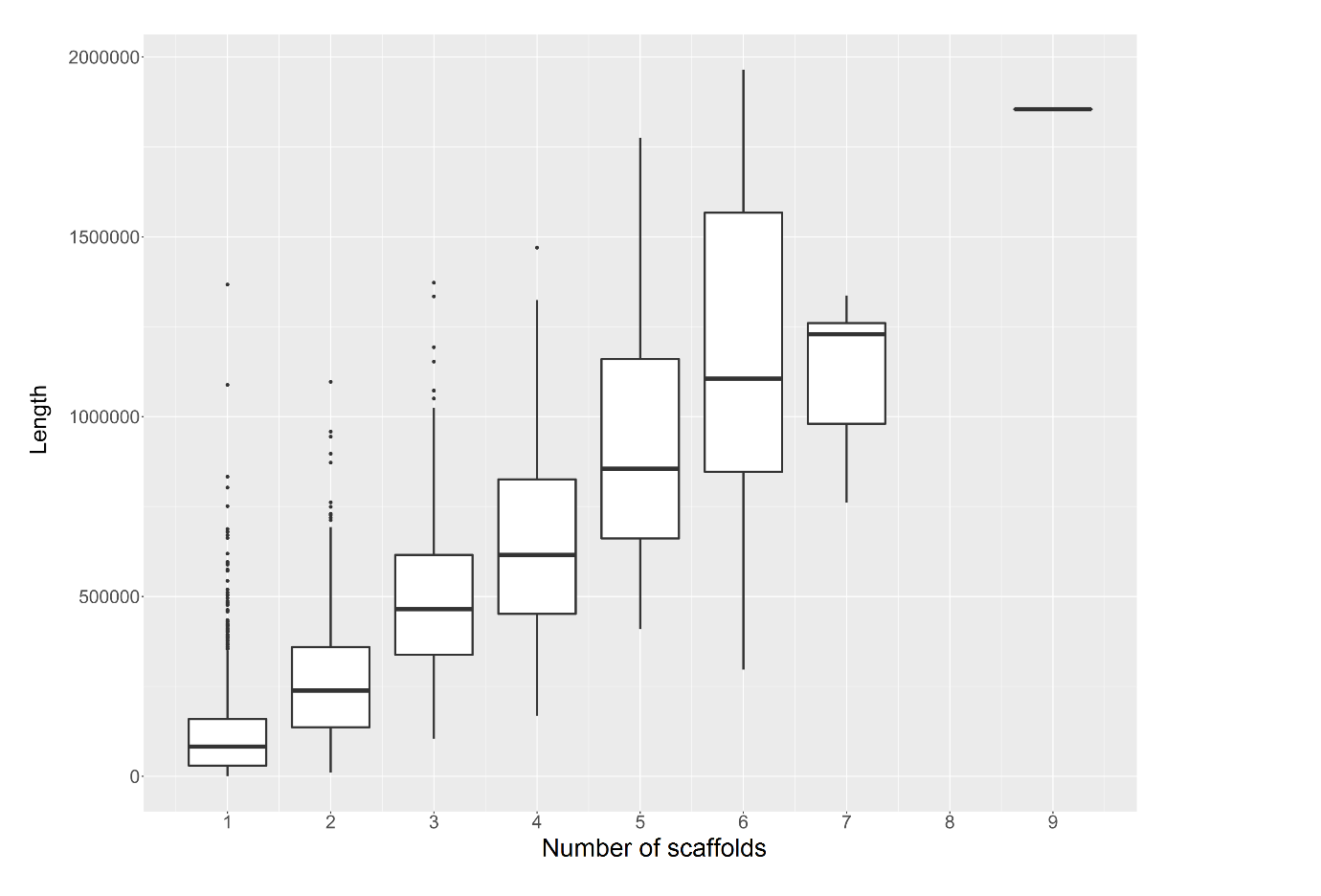

Supplement: Supplementary file 6 [file 1273FigureS1.docx]
