# Supplementary material for: A Genome-Wide Association Study for Host Resistance to Ostreid Herpesvirus in Pacific Oysters (Crassostrea gigas)
Source: G3 (Bethesda). 2018 Feb 22;8(4):1273–80. doi: 10.1534/g3.118.200113 (PMC5873916; doi:10.1534/g3.118.200113)
Supplement: Supplementary file 7 [file 1273TableS1.docx]

Table S1. Number of markers removed from each family due to missing genotypes and segregation distortion.

| Family | Progenies | Total | due to missing genotype | due to segregation distortion |
| --- | --- | --- | --- | --- |
| 1 | 2 | 534 | 435 | 99 |
| 2 | 2 | 2655 | 2569 | 86 |
| 3 | 4 | 502 | 325 | 177 |
| 4 | 4 | 806 | 388 | 418 |
| 5 | 4 | 744 | 349 | 395 |
| 6 | 5 | 891 | 334 | 557 |
| 7 | 5 | 675 | 342 | 333 |
| 8 | 5 | 751 | 343 | 408 |
| 9 | 5 | 946 | 503 | 443 |
| 10 | 5 | 943 | 486 | 457 |
| 11 | 6 | 1040 | 530 | 510 |
| 12 | 7 | 1050 | 512 | 538 |
| 13 | 13 | 3087 | 2482 | 605 |
| 14 | 33 | 4015 | 2452 | 1563 |
| 15 | 37 | 2700 | 473 | 2227 |
| 16 | 48 | 3853 | 502 | 3351 |
| 17 | 48 | 2657 | 418 | 2239 |
| 18 | 48 | 3162 | 780 | 2382 |
| 19 | 54 | 3192 | 485 | 2707 |
| 20 | 64 | 4085 | 377 | 3708 |
| 21 | 74 | 2513 | 605 | 1908 |
| 22 | 105 | 3112 | 468 | 2644 |
| 23 | 231 | 3800 | 380 | 3420 |

| Informative Markers | |  |
| --- | --- | --- |
| Maternally | | 21087 |
| Paternally | | 20528 |
| Both |  | 22136 |
